# *TCF7L2 *variant genotypes and type 2 diabetes risk in Brazil: significant association, but not a significant tool for risk stratification in the general population

**DOI:** 10.1186/1471-2350-9-106

**Published:** 2008-12-04

**Authors:** GF Marquezine, AC Pereira, AGP Sousa, JG Mill, WA Hueb, JE Krieger

**Affiliations:** 1 Heart Institute, University of São Paulo Medical School, Sao Paulo, Brazil; 2 Federal University of Rio Grande do Norte, Natal, Brazil; 3 Federal University of Espirito Santo, Vitória, Brazil

## Abstract

**Background:**

Genetic polymorphisms of the *TCF7L2 *gene are strongly associated with large increments in type 2 diabetes risk in different populations worldwide. In this study, we aimed to confirm the effect of the *TCF7L2 *polymorphism *rs7903146 *on diabetes risk in a Brazilian population and to assess the use of this genetic marker in improving diabetes risk prediction in the general population.

**Methods:**

We genotyped the single nucleotide polymorphisms (SNP) rs7903146 of the *TCF7L2 *gene in 560 patients with known coronary disease enrolled in the MASS II (Medicine, Angioplasty, or Surgery Study) Trial and in 1,449 residents of Vitoria, in Southeast Brazil. The associations of this gene variant to diabetes risk and metabolic characteristics in these two different populations were analyzed. To access the potential benefit of using this marker for diabetes risk prediction in the general population we analyzed the impact of this genetic variant on a validated diabetes risk prediction tool based on clinical characteristics developed for the Brazilian general population.

**Results:**

SNP rs7903146 of the *TCF7L2 *gene was significantly associated with type 2 diabetes in the MASS-II population (OR = 1.57 per T allele, p = 0.0032), confirming, in the Brazilian population, previous reports of the literature. Addition of this polymorphism to an established clinical risk prediction score did not increased model accuracy (both area under ROC curve equal to 0.776).

**Conclusion:**

*TCF7L2 *rs7903146 T allele is associated with a 1.57 increased risk for type 2 diabetes in a Brazilian cohort of patients with known coronary heart disease. However, the inclusion of this polymorphism in a risk prediction tool developed for the general population resulted in no improvement of performance. This is the first study, to our knowledge, that has confirmed this recent association in a South American population and adds to the great consistency of this finding in studies around the world. Finally, confirming the biological association of a genetic marker does not guarantee improvement on already established screening tools based solely on demographic variables.

## Background

Type 2 diabetes mellitus (T2D) is a heterogeneous disease characterized by different degrees of insulin resistance and defects in insulin secretion, both of which are thought to result from the interplay of genetic and environmental factors. The identification of causative genes that predispose to T2D could provide clues to better understand the primary pathogenesis and therefore result in better prevention, diagnosis and treatment of this increasingly prevalent and costly condition. Up until recently, results of research on the genetic field have been mostly elusive.

Genome-wide linkage scans have discovered chromosomic regions containing type 2 diabetes (T2D) susceptibility genes in chromosome 10q, which were later ascribed to intronic variations in the transcription factor 7-like 2 (*TCF7L2*) gene, which were strongly associated with a twofold increase in risk for T2D in an Icelandic population [1]. Two additional cohorts showed similar associations, giving a combined odds ratio (OR) of 1.6 (95% confidence interval (CI) 1.4 to 1.7, *p *= 4.7 × 10^-18 ^per allele). This finding has readily been replicated in cohorts of European [1-13], Asian [6,14-16] and African [6,17] descent, showing very similar results. Most evidence for *TCF7L2 *has come from case-control studies or intervention trials. There are much fewer studies that analyzed the effect of this polymorphism in the general population [10,18], but they suggest that diabetic carriers of the at-risk allele have more severe β-cell dysfunction and more microvascular complications but less metabolic syndrome features and a more protective lipid profile than non-carriers.

In addition, it is necessary to confirm this association in different populations worldwide. At moment, there are no studies in South American populations. Therefore, this study had two objectives: 1) investigate whether *TCF7L2 *variants are associated with diabetes in the Brazilian population; 2) evaluate how significant is the impact of this association in predicting the prevalent diabetes risk in the general population from Brazil.

## Methods

To respond the first objective, we used a cohort of patients from the MASS- II study and to address the second one, we used a sample representative from the general urban population of the city of Vitoria, Brazil, both of which are described below.

### Multi-vessel coronary artery disease patients (MASS II Study)

Six-hundred and eleven patients that have documented multi-vessel coronary artery disease and normal left ventricular function were included in the MASS II trial [19]. Here we have solely used information from the baseline characteristics of the studied population. Diabetes was not necessary for enrollment in this protocol. From these individuals we were able to obtain genotype data for *TCF7L2 *on 560 patients, of whom 190 had diabetes mellitus, based on American Diabetes Association criteria or previous treatment for diabetes. All patients gave written informed consent for participating in the study. The Ethics Committee of the Heart Institute approved the trial, and all procedures were performed in accordance with the Helsinki Declaration.

### General population of Vitoria/ES, Brazil

A cross-sectional study of risk factors for cardiovascular diseases was performed in the urban population of Vitoria, Brazil, using the WHO-MONICA project guidelines. The study design was based on cross-sectional research methodology and was developed by means of surveying and analyzing socioeconomic and health data in a probabilistic sample of residents aged 25 to 64 years from the municipality of Vitoria, ES, Brazil. The population was randomized and the sample was socioeconomically, geographically and demographically representative of the residents of this municipality. A selection of 2,268 residential homes located in Vitoria was made and these were visited. The project received approval from the Ethics Committee of the Biomedical Center of Universidade Federal do Espírito Santo (UFES). The selected individuals were asked to attend the Cardiovascular Investigation Clinic of the University Hospital for tests to be performed on the following day. Of the total sample, 1,577 individuals attended. Participants were submitted to physical examination. Major cardiovascular risk factors such as smoking habits, alcohol intake, sedentarism, diabetes and hypertension were inquired. Blood glucose, total cholesterol, lipoprotein fractions, and triglycerides were assayed by standard techniques in a 12 hour fasting blood sample.

Body mass index (BMI) (weight in Kg/height in meters^2^) was calculated, overweight defined as a BMI ≥ 25 Kg/m^2^, and obesity defined as a BMI ≥ 30 Kg/m^2^.

In order to screen individuals at higher risk for type 2 diabetes from the general population to be further investigated, we previously built a prediction model of diabetes risk in this same population (Sousa, AGP, personal communication). This simple risk score model was developed based in a population of 1,224 individuals from the general population without known diabetes aged 35 years or more. Also in this analysis, diabetes diagnosis was based on fasting glycemia equal or above 126 mg/dl (7 mmol/l). This model was externally validated in an independent population from a different city ascertained through a similar epidemiological protocol and items independently and significantly associated with diabetes, and chosen for inclusion in the model, were age, BMI and known hypertension. Selected variables in this model were age, BMI and hypertension.

The association between rs7903146 genotype and diabetes was measured using a genotype trend genetic model for an additive allelic effect captured by a regression model for diabetes in both population.

For model construction and performance measurement, univariate analyses were performed by logistic regression with T2DM (FPG > 126 mg/dl) as the dependent variable. Logistic regression coefficients were used to estimate odds ratios for each of the independent variables in the model. The continuous variables were categorized into strata. To create the final model, a score was calculated by multiplying the regression coefficient by 10 and rounding to the nearest integer. Finally, a sum score was calculated for each participant by adding the score for each significant variable in the risk model. Sensitivity, specificity, positive and negative predictive values and accuracy were compared for different score cutoffs. In addition, the proportion of subjects that needed subsequent testing (those individuals who have score values above the cutoff value) was compared between these several groups. In order to evaluate the model performance, a receiver operating characteristic (ROC) curve was built to plot probabilities saved with the logistic regression procedure and the area under ROC curve (AUC) was used to measure the power to discriminate high-risk from low-risk individuals.

### Genotyping of the rs7903146 polymorphism

Genomic DNA was extracted from peripheral blood leukocytes by means of a salting-out procedure. PCR primers used were: 5' ACA ATT AGA GAG CTA AGC ACT TTT TAG GTA 3' and 5' GTG AAG TGC CCA AGC TTC TC 3'. Briefly, the studied polymorphism was detected by polymerase chain reaction-restriction fragment length polymorphism assay (PCR-RFLP). A 30-cycle PCR was carried out in a PTC-DNA Engine Tetrad_2 _using a 10 μL reactive solution containing 10 mM Tris-HCl (pH 9.0), 50 mM KCl, 2.5 mM MgCl_2_, 100 μM of each dNTP, 0.3U *Easy Taq *DNA Polymerase, 5 pmol of each primer and 1 μL of genomic DNA template. PCR products were digested with 1U of *Rsa*I restriction enzyme and visualized by 3% agarose gel electrophoresis. The quality control for these assays was assessed by randomly selecting 40 samples that were re-genotyped by an independent technician. Observed concordance between genotyping assays was 100%.

### Statistical Analysis

We used SPSS v.13 program for statistical analysis. The goodness of fit for normal distribution was evaluated using the Kolmogorov-Smirnov test. To test for differences in various characteristics, non-parametric tests were used for continuous variables and the χ^2 ^test and Fischer exact test were used for categorical variables. Hardy-Weinberg equilibrium was studied through the use of Haploview software. For power calculation, we used the PS: Power and Sample Size Calculation from the Department of Biostatistics of Vanderbilt University, available for download at http://biostat.mc.vanderbilt.edu/twiki/pub/Main/PowerSampleSize/pssetup.exe. All statistical significance tests calculates are performed as a two-sided test and a value less than 0.05 was considered significant.

## Results

In the MASS-II population, a total of 560 individuals were genotyped for TCF7L2 gene variant rs7903146 and the genotypic distribution for this variant was in Hardy-Weinberg equilibrium. There was male sex preponderance (69.1%) and a high T2D prevalence of 31.0%. Mean BMI was 27.1 kg/m2. According to the used weight classification, 66.2% of this population was overweight or obese (Table 1). The relative frequency of genotypes CC, CT and TT in MASS II population was respectively 28,4%, 60,0% and 11,6% (number of individuals equal to 159, 336 and 65, respectively). Allele and genotype frequencies were not in Hardy-Weinberg equilibrium (p = 0,0004) (probably because of the high a priori chance of diabetic individuals in this sample with multi-vessel coronary artery disease). The rate of diabetes increased with an increasing dose of allele T of rs7903146 (OR = 1.57, 95%CI 1.16–2.11, p = 0.0032). These data are summarized in Table 3. After adjusting for other covariates potentially associated with T2D risk, presence of allele T was still significantly associated with a 1.61 (95%CI 1.18–2.19) increased risk of presenting T2D (p = 0.0025) (Table 4).

**Table 1:** Characteristics of the MASS-II population

	Total	rs7903146		p value
			
		CC/CT	TT	
***Number of patients (%)***	611 (100)	495 (88.4)	65 (11.6)	
Male	423 (69.1)	339 (88.5)	155 (88.1)	.88
Age (years)	59.8 ± 9.1	59.7 ± 9.12	58.8 ± 9.6	.45
***Mean BMI (kg/m^2^)***	27.1 ± 4.2	27.1 ± 4.2	27.1 ± 4.2	.97
< 25	201 (33.0)	160 (32.5)	19 (29.7)	.64
25 – 29.9	276 (45.4)	219 (44.4)	34 (53.1)	.18
≥ 30	129 (21.2)	112 (22.8)	10 (15.9)	.21
***Mean FPG (mg/dL)***	129.5 ± 58.2	127.21 ± 53.7	139.2 ± 75.6	.22
< 110	311 (52.2)	256 (52.7)	29 (45.3)	.26
110 – 125	107 (18.0)	90 (18.6)	12 (18.7)	.96
≥ 126	178 (30.0)	140 (28.8)	23 (36.0)	.24
***Total Cholesterol (mg/dL)***	223.2 ± 47.7	222.9 ± 47. 6	221.7 ± 52.5	.85
***Mean HDL (mg/dL)***	37.4 ± 10.4	37.5 ± 10.6	36.5 ± 9.9	.49
***Mean Triglycerides (mg/dL)***	195.1 ± 121.0	193.7 ± 118.0	179.9 ± 97.0	.37
				
	MS Components:			
***Obesity (BMI)***	129 (21.2)	112 (22.7)	10 (15.6)	.19
***High Triglycerides***	344 (57.1)	279 (57.1)	34 (53.1)	.55
***Low HDL-c***	447 (78.1)	371 (79.3)	46 (75.4)	.48
***Hypertension***	364 (59.6)	292 (59.1)	36 (55.4)	.64
***FPG ≥ 110 MG/dL***	278 (46.6)	223 (45.9)	35 (54.7)	.18

**Table 3:** Genotype association with type 2 diabetes

rs7903146		DM n (%)	Non-DM n (%)	OR (95%CI)	Allele test (P value‡)
**MS2**	CC	38 (22.0)	120 (31.1)	1.57 (1.16 – 2.11)	0.0032 (0.0034*)
	CT	106 (61.3)	230 (59.6)		
	TT	29 (16.8)	36 (9.3)		
**VIT**	CC	45 (40.2)	564 (43.6)	1.126 (0.84–1.51)	0.426
	CT	54 (48.2)	603 (46.6)		
	TT	13 (11.6)	128 (9.9)		

(*) Adjusted for age and sex.

(**‡**) additive genetic model

**Table 4:** Logistic regression for type 2 diabetes risk

	MASS II		Vitoria	
	
	OR (95%CI)	P value	OR (95%CI)	P value
T Allele	1.57 (1.18–2.19)	0.0025	1.15 (0.84–1.58)	0.391
Age	1.02 (0.99–1.04)	0.097	1.08 (1.06–1.10)	< 0.0001
Female Sex	0.27 (0.02–3.11)	0.29	1.04 (0.69–1.59)	0.837
Obesity*	1.67 (1.08–2.56)	0.021	4.91 (3.25–7.43)	< 0.0001

(*) BMI equal or greater to 30 kg/m^2^

In regards to the general population of Vitoria, 1,440 samples of DNA were available and genotyped for *TCF7L2 *rs7903146 variant. The characteristics of this population are shown in Table 2. Their mean age was 44.8 years (range 23–65). The prevalence of T2D was only 7.9%. Obesity was present in 19.3% of patients, but 55.3% were on the overweight category (BMI ≥ 25 kg/m^2^). Mean BMI was 26.2 ± 4.9 kg/m^2^. Non-diabetics individuals had a lower mean BMI (25.9 versus 30.6 kg/m^2 ^in diabetics). The prevalence of genotype CC was 43,4% (n = 614), genotype CT equal to 46,6% (n = 660) and overall prevalence of TCF7L2 genotype TT was 10,0% (n = 142). Allele and genotype frequencies were in Hardy-Weinberg equilibrium (p = 0,07) in this sample from the general population. A slightly higher genotype prevalence was observed in the diabetic sub-population of individuals (12.1%) as compared to non-diabetic individuals (9.9%), but this difference was not statistically significant (p = 0.46). Of particular interest, the total number of diabetics that carried the TT genotype, despite the relatively large size of the initial sample, came down to only 14 people. Therefore, the statistical power of detecting this association in the studied sample from the general population was only 24%.

**Table 2:** Characteristics of the Vitoria general population

	General	rs7903146		p
			
		CC + CT	TT	
Number of patients (%)	1577 (100)	1303 (89.9)	146 (10.0)	
Male	718 (45.6)	600 (46.0)	64 (43.8)	.61
Age (years)	44.8 ± 10.9	44.7 ± 10.9	44.3 ± 10.7	.65
Mean BMI (kg/m^2^)	26.3 ± 4.9	26.3 ± 5.0	26.2 ± 4.8	.82
< 25	692 (44.2)	573 (44.3)	65 (44.8)	.91
25 – 29.9	513 (35.7)	458 (35.5)	55 (38.0)	.54
≥ 30	285 (19.4)	260 (20.1)	25 (17.2)	.41
Mean FPG (mg/dL)	105.01 ± 32	105.0 ± 32.3	105.7 ± 29.3	.82
< 110	1130 (78.7)	1014 (78.3)	116 (80.0)	.64
110 – 125	241 (15.4)	200 (15.4)	22 (15.2)	.93
≥ 126	123 (7.8)	102 (7.9)	14 (9.7)	.45
Total Cholesterol (mg/dL)	214.4 ± 47.8	214.0 ± 48.1	215.4 ± 44.0	.74
Mean HDL (mg/dL)	45.4 ± 12.3	45.2 ± 12.1	44.0 ± 10.4	.27
Mean Triglycerides (mg/dL)	137.6 ± 127.9	137.2 ± 131.0	133.0 ± 82.1	.70
SBP (mmHg)	128 ± 2	128 ± 2	129 ± 2	.52
DBP (mmHg)	84 ± 1	84 ± 14	84 ± 14	.56
	MS Components			
Visceral obesity *	255 (16.2)	216 (16.6)	21 (14.4)	.50
High triglycerides	484 (30.7)	391 (30.1)	51 (35.0)	.23
Low HDL-c	848 (53.8)	705 (54.1)	79 (54.1)	.99
Hypertension	727 (46.1)	596 (45.7)	75 (51.4)	.19
FPG ≥ 110 MG/dL	334 (21.4)	281 (21.7)	29 (20.0)	.63
Metabolic syndrome	397 (25.4)	326 (25.2)	40 (27.6)	.53

*Abdominal circumference above cutoff points according to ATPIII criteria

**Figure 1:**
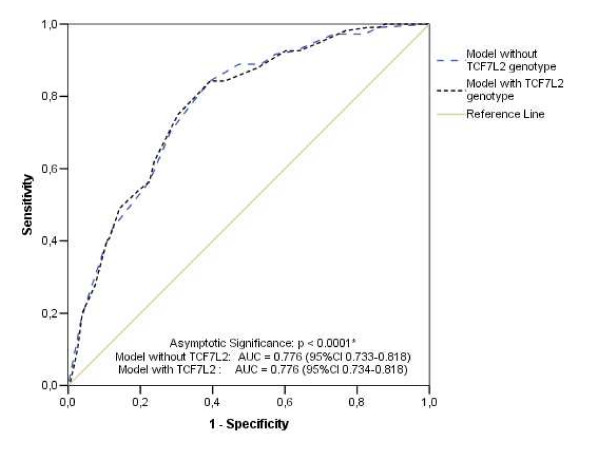
**ROC curves for the risk score in the two predictive models**. (*) p value for both models

Our previous risk model (Sousa AGP, unpublished results) based solely on clinical criteria (age, presence of hypertension and BMI) for predicting diabetes was able to reach a good performance to identify individuals with an increased likelihood of having diabetes. According to this tool, individuals classified as having a higher probability of becoming diabetic (higher score values) should be assessed with further laboratorial tests for diabetes. Interestingly, inclusion of the TT genotype in our model resulted in no improvement on the area under the ROC curve (AUC = 0.776; 95%CI 0.73–0.82 in both models). The performance characteristics of two models (with only demography variables and with *TCF7L2 *variables associated to demography variables) are shown in Table 5. There was a slight improvement in specificity when the *TCF7L2 *genotype TT was included, however the sensitivity diminished. There was almost no difference among the ROC curves of two models (Figure 1).

**Table 5:** Performance of two predictive models in assessment T2D risk

Model 1 – Only Demography variables								
Cutoffs Values	Sensitivity	Specificity	PPV	NPV	Accuracy	OR (95%CI)	P value	Needing to Additional Tests(%)
16.5	0.8426	0.6071	0.19	0.972	0.63	8.27 (3.86–12.68)	< 0.00001	43.7
18	0.7037	0.7162	0.213	0.957	0.71	5.99 (3.38–8.60)	< 0.00001	32.51
**Model 2 – Demography and TCF7L2 TT Genotype variables**								
Cutoffs Values								
16.5	0.8426	0.5939	0.185	0.972	0.62	7.83 (3.66–12.0)	< 0.00001	44.89
18	0.7222	0.7081	0.213	0.959	0.71	6.30 (3.51–9.1)	< 0.00001	33.42

Although several studies have observed that the rs7903146 SNP is associated with some of the metabolic syndrome features, we did not observe any significant association between the T risk allele and these characteristics (Table 1 and 2).

## Discussion

Recently Grant et al. [1] have described an association of common genetic variants in *TCF7L2 *gene and type 2 diabetes risk, a finding which has been promptly replicated in many populations worldwide and made this polymorphism one of the most important discoveries in the pathophysiology of T2D in decades [20-22]. Initially, we were able to confirm the association between polymorphism rs7903146 and T2D in a cohort of patients from the MASS II protocol, whose diabetes prevalence was 31.1%. To our knowledge, this is the first study that confirmed this association in a South American population. The odds ratio of association was similar to other previous studies in different populations [1,2,6,15].

In the second stage, we have genotyped a large sample of individuals from the general population of Brazil, which has gone through intense admixing in its recent history and is characterized by a genetic background that is remarkably heterogeneous when compared to cohorts of European or Asian ancestry. We genotyped a total of 1449 people looking for associations with diabetes and metabolic syndrome components, which also have been associated with this polymorphism in some, but not all, studied populations [8,10]. Even though genotype frequencies for the rs7903146 variants in our population were somewhat similar to those reported previously in different studies, we failed to confirm the association that has been showing great consistency in case-control [23] and general [11,24,25] populations all over the world. We believe this finding is mostly due to the lack of statistical power, because of the low diabetes prevalence and total number of diabetic patients in the tested population than to subtleties of our population such as ethnic variety and genetic heterogeneity.

Most importantly, however, the addition of a known *TCF7L2 *genotype in a validated model for predicting prevalent diabetes was not able to improve the performance of the risk score: area under ROC curve in both models equal to 0.776, ie the inclusion of *TCF7L2 *genotype is not better than the utilization of only clinical characteristics to predict diabetes risk in our population. A very similar result has been recently published by Balkau *et al *[26] in an incidendt diabetes study in the French population. The clinical and laboratorial prediction model improved only modestly after inclusion of TCF7L2 and interleucin 6 (IL-6) SNPs information. Another example of a practical consequence of applying the predictive model is the value of Needing to Additional Tests (Table 5), which means the proportion from population which would be tested for T2D according to the model. It was observed only a little difference in percentage of individuals that will be further tested according to both models. In addition, as has been demonstrated, the impact of most polymorphisms on determining T2D risk is only modest. In order to improve the accuracy of clinical predictors to assess diabetes risk in a general population, it might be necessary to add several genetic variants in the model [27,28]. Recent work from van Hoek *et al *[28] combined information from 18 polymorphisms which have have been related to T2D and tested them in a general population of elderly persons. The prediction power is modestly enhanced when a model with genetic information is compared to a clinical characteristics model.

Finally, we also looked for a link between the rs7903146 variant of the *TCF7L2 *gene and features of the metabolic syndrome such as weight, body mass index, hypertriglyceridemia, hypertension, low HDL-c, as reported earlier [8,18], but could not observe this even when data were adjusted for age, race and abdominal circumpherence.

## Conclusion

This finding adds to the importance of this polymorphism in the pathogenesis of the disease and to the great consistency in which data are replicated around the world. The use of this particular genetic marker in stratifying individuals regarding the risk of T2D appears to be dependent on the *a priori *risk of the disease in the tested population. One should exercise caution in the indiscriminate use of genetic association results once the confirmation of the biological association of a genetic marker with a common chronic disease does not guarantee meaningful improvement on already established screening tools based solely on demographic variables.

## Competing interests

The authors declare that they have no competing interests.

## Authors' contributions

WH, JGM, and ACP performed the field work. GFM, AGPS, and ACP performed the statistical analyses. GFM, AGPS, and ACP drafted the manuscript under the supervision of WH, JGM and JEK. ACP supervised the study. All authors read and approved the final manuscript.

## Pre-publication history

The pre-publication history for this paper can be accessed here:

http://www.biomedcentral.com/1471-2350/9/106/prepub
